# A Peptide Mimetic Targeting *Trans*-Homophilic NCAM Binding Sites Promotes Spatial Learning and Neural Plasticity in the Hippocampus

**DOI:** 10.1371/journal.pone.0023433

**Published:** 2011-08-24

**Authors:** Igor Kraev, Christian Henneberger, Clara Rossetti, Lisa Conboy, Lene B. Kohler, Martina Fantin, Alistair Jennings, Cesar Venero, Victor Popov, Dmitri Rusakov, Michael G. Stewart, Elisabeth Bock, Vladimir Berezin, Carmen Sandi

**Affiliations:** 1 Department of Life Sciences, The Open University, Milton Keynes, United Kingdom; 2 Department of Clinical and Experimental Epilepsy, Institute of Neurology, University College London (UCL), London, United Kingdom; 3 Laboratory of Behavioral Genetics, Brain Mind Institute, EPFL, Lausanne, Switzerland; 4 Protein Laboratory, Department of Neuroscience and Pharmacology, Panum Institute, University of Copenhagen, Copenhagen, Denmark; 5 Department of Psychobiology, UNED, Ciudad Universitaria, Madrid, Spain; University of Minnesota, United States of America

## Abstract

The key roles played by the neural cell adhesion molecule (NCAM) in plasticity and cognition underscore this membrane protein as a relevant target to develop cognitive-enhancing drugs. However, NCAM is a structurally and functionally complex molecule with multiple domains engaged in a variety of actions, which raise the question as to which NCAM fragment should be targeted. Synthetic NCAM mimetic peptides that mimic NCAM sequences relevant to specific interactions allow identification of the most promising targets within NCAM. Recently, a decapeptide ligand of NCAM—plannexin, which mimics a homophilic trans-binding site in Ig2 and binds to Ig3—was developed as a tool for studying NCAM's *trans*-interactions. In this study, we investigated plannexin's ability to affect neural plasticity and memory formation. We found that plannexin facilitates neurite outgrowth in primary hippocampal neuronal cultures and improves spatial learning in rats, both under basal conditions and under conditions involving a deficit in a key plasticity-promoting posttranslational modification of NCAM, its polysialylation. We also found that plannexin enhances excitatory synaptic transmission in hippocampal area CA1, where it also increases the number of mushroom spines and the synaptic expression of the AMPAR subunits GluA1 and GluA2. Altogether, these findings provide compelling evidence that plannexin is an important facilitator of synaptic functional, structural and molecular plasticity in the hippocampal CA1 region, highlighting the fragment in NCAM's Ig3 module where plannexin binds as a novel target for the development of cognition-enhancing drugs.

## Introduction

Neural cell adhesion molecule (NCAM), a prominent protein of the immunoglobulin (Ig) superfamily, plays a key role in neural development and synaptic plasticity. NCAM is expressed as three major isoforms with molecular weights of 180, 140 and 120 kDa. Extracellularly, all NCAM isoforms contain five Ig domains and two fibronectin type 3 (F3) domains [1]. The mechanisms by which NCAM affects neural plasticity include activation of intracellular signaling cascades and regulation of cell–cell and cell–extracellular matrix adhesion and de-adhesion [2]. De-adhesion processes are mediated largely by the addition of long sialic acid residues (polysialic acid, PSA) to NCAM's Ig5 domain [3], whereas adhesion processes involve both homophilic and heterophilic interactions of NCAM [4].

A key role for NCAM in memory formation has been established through animal studies showing that NCAM expression transiently increases in the hippocampus after training in spatial or contextual tasks [5], [6]. Interfering with the function or expression of NCAM through pharmacological or genetic approaches impairs memory function and long-term potentiation [7]–[10]. This evidence implicates NCAM as a particularly relevant target for the development of cognition-enhancing drugs. Given NCAM's structural and functional complexity it is critical to first identify the most promising target within NCAM. This has recently been achieved by developing synthetic NCAM mimetic peptides that mimic NCAM sequences relevant to specific interactions based on combinatorial chemistry and structural studies [4]. Mimicking NCAM's interaction with the fibroblast growth factor receptor (FGFR) through the pentadecapeptide FGL, which corresponds to the FGFR-binding region in F3II of NCAM, has been very successful in improving cognition [11], [12]. FGL can affect behavior in the absence of NCAM [13].

The crystal structure of an NCAM fragment combining the first three Ig modules revealed NCAM's homophilic binding mechanisms. The pattern of specific interactions between these Ig modules indicated that Ig1 and Ig2 mediate dimerization of NCAM molecules expressed on the same cell surface (*cis*-interactions), whereas Ig3 mediates interactions between NCAM molecules on the surfaces of opposing cells (*trans*-interactions) by simultaneously binding to Ig1 and Ig2 [14]. Although the cognitive effects of tackling NCAM *cis*-interactions have already been reported [15], the effects of mimicking NCAM *trans*-interactions remain unexplored. Recently, a decapeptide ligand of NCAM—plannexin, which mimics a homophilic trans-binding site in Ig2 and binds to Ig3— was developed as a tool for studying NCAM's *trans*-interactions [16]. This peptide promotes neurite growth and cell survival *in vitro*. Here, we have found that plannexin promotes neurite outgrowth in hippocampal cell cultures and spatial learning both under normal conditions and under PSA deficiency. Plannexin also facilitated synaptic transmission in the CA1 hippocampal region. Moreover, an increase in the density of mushroom-like dendritic spines and in the synaptic incorporation of AMPA glutamate receptors was observed in CA1.

## Materials and Methods

### Ethics Statement

All procedures were conducted in accordance with the Swiss National Institutional Guidelines of Animal Experimentation and were approved by the Swiss Cantonal Veterinary Office Committee for Animal Experimentation (Licenses number 1647.1 and 1747.4).

### Peptides

The plannexin peptide (DVRRGIKKTD) consists of a combination of sequences from the CD (three amino acids) and EF loops (seven amino acids) of the NCAM Ig2 module that, according to the crystal structure, are involved in homophilic contact with the Ig3 module [16]. The name plannexin is given to the peptide because it is derived from the binding site in the NCAM Ig2 module where it binds to the NCAM Ig3 module in trans-homophilic interactions of two NCAM molecules in the so-called flat (plane) zipper formation. The identification of the peptide (with this name) has been published previous [16]. Plannexin and a corresponding scrambled peptide (KTKVDRDGRI) were purchased from Schafer-N (Copenhagen Denmark). The peptides were synthesized as tetrameric dendrimers (plannexin-d) composed of four monomers coupled to a three-lysine-containing backbone. The peptides were dissolved in sterile distilled water or CSF. The concentrations of the peptides were measured spectrophotometrically by absorption at 205 nm.

### Materials for cell culture

Endoneuraminidase-N (EndoN) was a kind gift from Prof. Rita Gerardy-Schahn (Hannover, Germany). Polyclonal rabbit anti-GAP-43 antibodies were obtained from Chemicon (Temecula, CA, USA). Monoclonal anti-PSA-NCAM mouse antibody was obtained from AbCys S.A. (Paris, France) Alexa Fluor 488 polyclonal goat anti-rabbit and Alexa Fluor goat anti mouse 547 antibodies were obtained from Molecular Probes (Eugene, OR, USA). NeurobasalTM medium, basal modified Eagle's (BME) medium, fetal calf serum (FCS), penicillin, streptomycin, B27 supplement, glutamax, Na-pyruvate, and HEPES were purchased from Gibco BRL (Paisley, UK). Trypsin, DNAse 1, soybean trypsin inhibitor, bovine serum albumin (BSA), cytosine β-D-arabino-furanoside (AraC) and poly-L-lysine (PLL) were from Sigma-Aldrich Denmark A/S (Copenhagen, Denmark). Antifade fluorescence mounting medium was from Dako (Glostrup, Denmark). All tissue culture plastic was obtained from NUNC (Roskilde, Denmark).

### Cell cultures

Cells were routinely incubated at 37°C in a humidified atmosphere containing 5% CO_2_. Cerebellar granule neurons (CGNs) were obtained from postnatal day 3 Wistar rat pups (Charles River, Sulzfeld, Germany) essentially as previously described by Schousboe et al. [17]. Briefly, after dissection, the cerebella were cleared of blood vessels and meninges, crudely homogenized by chopping before trypsin treatment, and washed in the presence of DNase and trypsin inhibitor. Cellular debris was then pelleted by centrifugation. The CGNs were plated in Neurobasal medium supplemented with 0.4% w/v BSA, 2% v/v B27, 20 mM HEPES, 1% v/v glutamax, 100 U/ml penicillin, and 100 µg/ml streptomycin. Hippocampal neurons were obtained from embryonic day 19 (E19) Wistar rat embryos (Charles River Laboratories, Sulzfeld, Germany). The embryos were decapitated, and the brains were removed. Hippocampi were dissected and cleared of membranes and blood vessels in ice-cold modified Krebs-Ringer buffer. Cultures of single neurons were prepared as described above for CGNs.

### Neurite outgrowth assay

Dissociated neurons were plated at a density of 12,500 cells/cm^2^ directly on plastic in eight-well Permanox Lab-Tek chamber slides in Neurobasal™ medium supplemented as described above with or without peptides. For enzymatic removal of PSA from NCAM, cultures were treated with 15 nM (CGNs) and 30 nM (hippocampal neurons) of the enzyme endoneuraminidase (EndoN). Twenty-four hours later, the cells were fixed and immunostained for GAP-43 to visualize the neurons. Images of at least 200 cells were captured for each group in each individual experiment in a systematic series of fields of view as previously described [18] by computer-assisted fluorescence microscopy using a Nikon plan 20× objective (Nikon, Tokyo, Japan) and a video camera (Grundig Electronics, Germany). The average neurite length per cell was estimated using a stereological approach [18] and the Prima software package developed at the Protein Laboratory (Copenhagen, Denmark).

### Subjects

Rats and mice were used throughout the study. The rats were male Sprague-Dawleys (Charles River, France) weighing 275–300 g upon arrival. Wild-type (WT) mice or knock-out (KO) mice for the polysialyltransferase (PST; ST8SiaIV) gene (PST KO) were used. Experiments always included age-matched WT littermates (between 4 and 8 months old at the onset of behavioral testing). These animals were obtained from our animal house by intercrossing heterozygous mice to obtain homozygous KO mice and WT littermates. All mice originated from different PST breeding couples, which had been previously backcrossed for more than 10 generations into the C57BL/6J background. All animals were housed in groups of three per cage under light- (12 h/12 h light/dark cycle; lights on at 07:00 h) and temperature- (22±2°C) controlled conditions. Food and water were available *ad libitum*. Behavioral experiments were conducted between 09:00 h and 14:30 h.

### Genotyping

All mice were genotyped before and after behavioral testing by polymerase chain reaction (PCR). Tail DNA was extracted with a GenScript TissueDirect Multiplex PCR System (GenScript Corporation, Piscataway, NJ, USA) and analyzed by PCR in buffer containing 1.0 U/50 µl Taq DNA Polymerase, 45 mM KCl, 2.5 mM Mg2+, 200 µM dNTP (Eppendorf Hotmastermix, Eppendorf AG, Hamburg, Germany) and 0.4 µM of the corresponding primers (for PST, the forward primer for WT and KO mice was 5_-GAG CTC ACA ACG ACT CTC CGA GC-3 and the reverse primers were 5_-CTC AGT TCT GGC TAT TTC TTT TGT-3_ and 5_-ACC GCG AGG CGG TTT TCT CCG GC-3, respectively. The cycling conditions for the PST PCR were 95°C for 3 min, 56°C for 1 min and 72°C for 2 min for the first cycle. This was followed by 35 cycles at 95°C for 1 min, 56°C for 1 min and 72°C for 2 min, with a final extension at 72°C for 10 min. PCR products were resolved on a 2% agarose gel to determine genotypes (PST was 533 bp for WT and 730 bp for KO).

### Field potential recordings

Acute 350-µm-thick hippocampal slices from three- to four-week-old male Sprague-Dawley rats were transferred to a recording chamber (Scientific Systems Design, NJ), superfused with (mM) 119 NaCl, 2.5 KCl, 2 CaCl_2_, 1.3 MgSO_4_, 26 NaHCO3, 1 NaH_2_PO_4_, and 10 glucose at room temperature and bubbled with 95% O_2_/5% CO_2_. The slice cutting procedure has been previously described in detail [19]. Field potentials (fEPSPs) were recorded through glass electrodes filled with bath solution positioned either in the stratum moleculare of the dentate gyrus or in the stratum radiatum of the CA1 area. Field responses were evoked by electrical stimulation of the perforant pathway or Schaffer collaterals using mono- or bipolar stimulation electrodes (100 µs, 10–120 µA). They were then recorded (Multiclamp 700B, Molecular Devices, Sunnyvale, U.S.A.), sampled (National Instruments, Austin, U.S.A.) and stored for off-line analysis (WinWCP, John Dempster, University of Strathclyde, UK). Responses were elicited every 30 sec. Baseline responses were recorded for at least 10 min before plannexin or its scrambled variant were bath-applied for 30 min followed by a wash-out period. The initial slope of the fEPSPs was analyzed.

### Spatial learning in the water maze

The water maze was a circular pool. For rat experiments, the pool was black (2 m diameter, 45 cm high) and filled with normal water (30 cm depth). For mouse experiments, the pool was white (140 cm diameter) and filled with opaque colored water. The water temperature in all experiments was set at 25±1°C. An invisible escape platform (11 cm diameter for rats and 10×10 cm for mice) was placed at a fixed location equidistant from the sidewall and the middle of the pool and submerged 1.3 cm (mice) or 1.5 cm (rats) below the water's surface. The respective water mazes were surrounded by grey curtains placed at least 25 cm from the pool's periphery. The curtains had several prominent visual cues. Behavior was monitored by a video camera mounted in the ceiling above the center of the pool. The camera was connected to a computerized video tracking system (Ethovision 3.1.16, Noldus IT, Wageningen, The Netherlands). Mice were habituated to the room, apparatus, and water by giving them a 1 min free-swim trial one day before training. Spatial learning sessions were conducted on 3 consecutive days (days 1–3) with 4 trials per day. Each trial started by introducing the animal into the maze at one of 4 possible positions that were randomly balanced between trials and days. The latency and cumulative distance that animals needed to swim to find the hidden platform were measured. If an animal did not find the platform within 90 sec (rats) or 60 sec (mice), it was gently guided toward it. Each animal was allowed to remain on the platform for 30 sec before it was returned to its waiting cage. After the last trial in each session, the animals were first carefully dried with a towel and then placed in a waiting cage heated to 32°C for 10 min. The animals were then returned to their home cages. On day 4, a 60 sec probe test with no platform present was performed. The animals were given drug treatments immediately after water maze training on days 1 and 2. Since animals were only started to be treated after training on day 1, data for water maze training is analyzed and represented starting on day 2.

### Acoustic fear conditioning

Rat training and testing took place in three identical rodent observation cages (30×37×25 cm) placed in a sound-attenuating chamber illuminated by a 20 W bulb. The side walls of the observation cages were constructed of stainless steel, and the door was Plexiglas. The floor consisted of 20 steel rods wired to a shock source and a solid-state scrambler for the delivery of footshock unconditioned stimuli (US). Ventilation fans provided a background noise of 68 dB (whole system: Panlab, Spain). The animals were transported from the colony room to the adjacent fear-conditioning room where training and testing occurred. For training, rats were exposed to Context A (black walls of smooth texture, steel grid floor cleaned with 2% ethanol) for 160 s, followed by three presentations of tone-shock pairings in which the tone (20 s, 80 dB sound at 1000 Hz) co-terminated with a foot shock (0.4 mA, 1 s). The inter-tone interval was 40 s, and the conditioning session lasted 5.5 min in total. In the tone memory test (performed 24 h after the AFC session), rats were put into Context B (green walls of rough texture, grey plastic floor covered with flocks cleaned with 1% acetic acid) for 8 min in total and confronted with the same tone used in training during the last 5 min. Freezing, defined as behavioral immobility except for respiratory movements, was scored every 30 s and transformed into a percentage value. Animals were treated with the peptide plannexin or vehicle immediately after the ACF training session.

### Elevated plus maze, open field and novel object reactivity

To study the effects of drug treatment on anxiety-like behaviors and on exploratory activity, we tested rats in the open field (10 min), and 2 h later, we tested them in the elevated plus maze (EPM) for 5 min. Rats received two plannexin injections (2.5 µg/5 µl) on the two days preceding the behavioral test day (one injection/day). In all tests, behavior was monitored using a video camera and analyzed with a computerized tracking system (Ethovision 3.1.16, Noldus IT, The Netherlands).

Anxiety-related behaviors were evaluated using the EPM test [20]. Briefly, the plus maze consists of two opposing open arms (45×10 cm) and two enclosed arms (45×10×38 cm) that extend from a central platform (10×10 cm) elevated 50 cm above the ground. Light was adjusted to levels of 10–12 lx in the center of the maze. The rats were placed individually on the central platform and allowed to explore the maze for 5 min. Time spent in the open and closed arms was recorded. Anxiety was assessed as the time spent in the open arms.

Exploratory activity was tested in the open field test. The open-field consisted of a black pool with a diameter of 1 m and a depth of 40 cm. The floor of the pool was divided into three zones: an outer zone with a diameter of 1 m, an inner zone with a diameter of 75 cm, and a center zone with a diameter of 25 cm. Light was adjusted to a level of 8–10 lx in the center of the pool. Animals were placed in the center of the pool and their open-field activity was recorded for 10 min.

### Surgery, drug infusions, and control for cannula placement

The rats and mice underwent surgery for i.c.v. cannulation. They were anesthetized intraperitoneally with xylazine (10 mg/kg) and ketamine (80 mg/kg). A 22-gauge (rats) or 26-gauge (mice) metal guide cannula (Plastics One, Roanoke, VA, USA) fitted with a removable dummy cannula was stereotactically implanted above the right lateral cerebral ventricle (rats: 1.0 mm posterior, 1.5 mm lateral from bregma, and 4.3 mm ventral from the skull surface; mice: 0.36 mm posterior, 1.0 mm lateral from bregma and 2.0 mm ventral from the skull surface). The coordinates were based on the atlases of Paxinos and Watson [21] (rats) and Paxinos and Franklin (mice). The cannula was fixed to the skull with a cyanoacrylate adhesive (Vetbond, 3M, St. Paul, Minnesota, USA) and dental cement (Paladur, Hereaus-Kulzer). After surgery, the animals were housed individually and allowed at least 1 week to recover from the surgery. For i.c.v. injections, the dummy cannula was removed and replaced with an infusion cannula (Plastics One) 0.5 mm longer than the guide cannula and attached to a 10 µl Hamilton syringe via polyethylene tubing. A total volume of 5 µl (rats) or 1 µl (mice) of solution was injected with a micro-injection pump (Harvard apparatus, Cambridge, MA) with an infusion rate of 1–0.25 µl/min. The infusion cannula was left in place for an additional 2 min to allow for diffusion. Plannexin and the scrambled peptide were i.c.v. infused, both in rats and mice, at a dose of 2.5 µg/animal that was chosen based on the in vitro data and our experience finding the corresponding effective dose for in vivo application accumulated with other NCAM-derived memory enhancing peptides derived [11], [15]. The peptide concentration delivered in a 5 µl volume was 97 µM (i.e., 0.485 nmol).The PSA-cleaving enzyme EndoN was administered to rats at a dose of 0.25 U/injection 24 h before the start of water maze training. Peptides and EndoN were diluted in sterilized artificial cerebrospinal fluid (CSF), pH 7.4. Solutions were freshly prepared before injection.

Following the behavioral tests, the animals were anesthetized with sodium pentobarbital (50 mg/kg, i.p.). A cresyl violet solution was administered following the same protocol as for the drug injections. After decapitation, brains were removed and examined for the presence of cresyl violet. Only animals with a correctly placed cannula were included in the analyses.

### Immunocytochemistry

To verify the effectiveness of EndoN treatment, rats injected with plannexin and vehicle were deeply anesthetized with sodium pentobarbital and transcardially perfused with 100 ml of phosphate-buffered saline (PBS; pH = 7.4) followed by 250 ml of 4% paraformaldehyde in 0.1 M phosphate buffer. Brains were postfixed in the same fixative solution for 4 h. Coronal sections (50 µm thick) were cut on a vibratome (Leica VT 1000S; Glattbrugg, Switzerland) and collected in PBS. Briefly, brain sections were pre-washed in PBS (three times for 10 min each) and incubated with 3% H_2_O_2_ in PBS for 10 min to block endogenous peroxidase activity. After being washed in PBS, the sections were treated for 1 h with 10% normal donkey serum (NDS, Jackson ImmunoResearch Laboratories, Basel, Switzerland) and 0.2% Triton X-100 (Sigma, Buchs, Switzerland) in PBS. After being washed in PBS, they were incubated for 48 h at 4°C with monoclonal mouse anti-Men PSA-NCAM antibody (AbC0019; Abcys, Paris, France) at a dilution of 1∶1500. PBS containing 0.2% Triton-X-100 and 5% NDS was used for primary and secondary antibody dilutions. After washing in PBS, the sections were incubated for 1 h at room temperature (RT) with a biotin-SP-conjugated donkey anti-mouse immunoglobulin M (IgM) (Jackson ImmunoResearch Laboratories, Basel, Switzerland) at a 1∶200 dilution. Finally, a 1∶300 dilution of streptavidin-horseradish peroxidase conjugate (GE Healthcare UK Limited, Zurich, Switzerland) was added and incubated for 1 h at RT. PSA-NCAM antibody-peroxidase complexes were visualized using 0.5 mg/ml diaminobenzidine (DAB; Sigma- Aldrich, Basel, Switzerland) and 0.01% H_2_O_2_ in PBS. The sections were mounted on superfrost slides, dehydrated with an ethanol series, and coverslipped with DPX (Sigma-Aldrich, Basel, Switzerland). For the quantification of PSA-NCAM immunoreactivity, 5 coronal hippocampal sections from each rat (taken between 3.55 and 3.8 mm posterior to bregma) were processed. The total number of PSA-NCAM immunoreactive cells in the dentate gyrus of each slice was manually counted in both hemispheres under a microscope with a 40× objective.

For immunohistochemical characterization in electron microscopy experiments, the 50-µm hippocampal sections were blocked in incubation buffer for 2 hours, followed by incubation in primary antibody (mouse anti-PSD-95, 1∶400 and rabbit anti-PSD-93, 1∶200) in incubation buffer overnight at 4°C. After being rinsed in PBS, the sections were incubated in secondary antibody (donkey anti-rabbit, 1∶100 and donkey anti-mouse, 1∶100) in incubation buffer for 4 h at RT. After another set of washes, ABC Elite reagent (Vector Laboratories, UK) was applied for 1 h. As substrate for the peroxidase reaction, diaminobenzedine (DAB, Sigma, USA) was applied for 5 min at a concentration of 0.22 mg/ml in Tris buffer (pH 7.4) with 0.01% hydrogen peroxide. The sections were thoroughly washed and prepared for electron microscopy.

### Electron Microscopy

Rats were anesthetized with pentobarbital and transcardially perfused with 3.75% acrolein/2% paraformaldehyde (in 0.1 M phosphate buffer) following water maze training; more precisely, on day 4, after submitting animals to the probe trial. The hippocampi were then coronally sectioned with a vibratome at 50 um and processed for electron microscopy as described previously (1). Ultrathin serial sections were taken from the stratum radiatum of area CA1 of the hippocampus, stained, and examined in a JEOL 1010 electron microscope. Electron micrographs were taken at 6000× magnification. Cross-sectioned myelinated axons, dendrites, and mitochondria spanning all sections provided a fiduciary reference for the initial alignment of serial sections. Section thickness was determined using the approach of Fiala and Harris (http://synapse-web.org). For digital reconstructive analyses, digitally scanned EM negatives were aligned as JPEG images (software available from Fiala and Harris: http://synapses.clm.utexas.edu/tools/index.stm) and contours of individual cells, and their elements were traced digitally and computed.

Stereological estimates of synapse density in the stratum radiatum of CA1 area were made 90–110 µm from the proximal edge of the CA1 pyramidal cell layer. Stereological analyses were performed as previously described [22], [23] on tissue volumes of approximately 500–800 mm3. Synapses were identified via postsynaptic densities (PSDs) and the presence of at least two presynaptic vesicles). The synapses were then categorized according to [24], [25]. A spine is classified as a *mushroom* if its head is significantly wider than the width of its neck; as *thin* if its length is greater than the width of its neck and head; and *stubby*, if the width of the neck is similar to its length. Generally, the volume of a thin spine is approximately 10 times less than that of a mushroom spine.

The synaptic number was counted within these areas irrespective of the presence of components such as large dendrites and non-spiny dendrites of interneurons, in order to avoid bias in the data obtained. Synaptic densities were expressed as number of synapses per 100 µm3 of tissue.

### Preparation of hippocampal homogenates and synaptoneurosomes

Synaptoneurosomes (SNS) were prepared according to the method described by Quinlan et al. [26] and Conboy and Sandi [27]. Briefly, the CA1 area of the hippocampus was homogenized in ice-cold homogenization buffer (10 mM HEPES/1.0 mM EDTA/2.0 mM EGTA/0.5 mM DTT/0.1 mM PMSF) containing a protease and phosphatase inhibitor cocktail (Roche, Switzerland). The homogenates were then passed through two 100-µm-pore nylon mesh filters. At this stage, aliquots of whole hippocampus were taken, solubilized with 1% NP-40, and stored at −80°C for future analyses. The remaining tissue was filtered twice through 5-µm-pore filters. Filtered homogenates were centrifuged at 3600× *g* for 10 min at 4°C. The resultant pellets were re-suspended in 100 µL 1% SDS, boiled for 10 min, and stored at −80°C.

### Quantification of FGFR1 phosphorylation

Phosphorylation of FGFR1 was quantified in hippocampal homogenates by an enzyme-linked immunoabsorbent assay (ELISA). In brief, flat-bottom 96-well microplates were allowed to adsorb a coating solution (Na_2_CO_3_ 0.1 M/NaHCO_3_, 0.1 M) for 2 h at RT. The solution was removed, and 50 µl of pellet sample was added at a concentration of 10 µg/ml to each well of the polystyrene flat-bottom ELISA plate. Plates were incubated overnight at 4°C and then washed three times with 0.03 M PBS containing 0.05% Tween 20, pH 7.4. Additional binding sites were blocked with BSA (3%) for 2 h at RT. The wells were rinsed three times as described above and incubated with 50 µl aliquots of polyclonal rabbit anti-FGFR1 phosphospecific antibody (1∶100, Biosource International, Inc. USA) for 20–24 h at 4°C. Then, the wells were washed, and 50 µl aliquots of anti-rabbit IgG peroxidase conjugate (whole molecule conjugate; 1∶500; Sigma, Spain) were added for 2 h. Subsequently, 50 µl of citrate buffer (50 mM Na_2_HPO_4_, 25 mM citric acid, pH 4.5) containing 1 mg/ml *o*-phenylene diamine and 0.06% H_2_O_2_, added just before use was placed in each well. The peroxidase was allowed to react for 10 min at RT. The reaction was terminated by the addition of 50 µl of 5 N H_2_SO_4_ to each well. The optical density was determined by measuring absorbance at 492 nm with a Microplate Reader (DigiScan Reader V3.0 and DigiWIN software Program; ASYS Hitech GmbH, Austria).

### Quantitative western blotting

Protein content in whole and synaptoneurosome hippocampal samples was quantified using the DC protein assay (Biorad Laboratories AG, Switzerland). Quantitative western blotting was carried out as previously described [23]. Briefly, equal protein samples were prepared at a concentration of 0.5 µg/ml in 33 mM NaCl, 70 mM Tris-HCl, 1 mM EDTA, 2% (w/v) SDS, 0.01% (w/v) bromophenol blue, 10% glycerol, pH 6.8. Proteins were resolved on 10% polyacrylamide gels and transferred to nitrocellulose membranes. Antibodies against GluA1 (1∶10000, Assay Design, MI), GluA2 (1∶2000, AbCAM, UK), GluA3 (1∶1000, Invitrogen AG, Switzerland), NR1 (1∶10000, Millipore, Switzerland) and actin (1∶20000, Sigma-Aldrich, Switzerland) were applied and detected within the linear range of detection. Immunoreactive bands were detected using the Biorad ChemiDoc XRS system. Densitometric analysis was calculated using Biorad Quantity One (4.2.3) software (Biorad Laboratories AG, Switzerland). The absorbance for each of the synaptic protein antibodies was normalized to within-lane actin absorbance. Average densitometric data are reported for plannexin as a percentage of the vehicle control values.

### Statistics

Statistical analyses were performed using GraphPad Prism v5.03 (GraphPad, San Diego, CA, USA) and SPSS, and they involved a preliminary assessment of data normality with the D'Agostino-Pearson test. When normality was confirmed, the data were analyzed with either Student's t-test or ANOVA (either factorial, one-way or repeated measures) followed by Newman-Keuls or the Bonferroni *post hoc* test, when appropriate. When normality was rejected, the data were analyzed using the nonparametric Mann-Whitney U test. Significance was set at p<0.05.

## Results

### Plannexin induces neurite outgrowth in primary neuronal cultures that is independent of PSA expression

**Figure 1:**
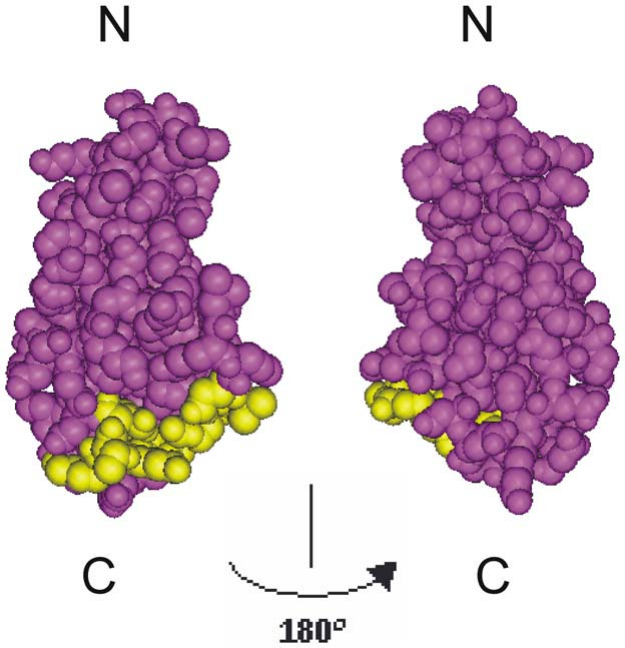
Localization of the plannexin sequence in the NCAM Ig module. A space-filling model of Ig2 with two 180° rotation projections (margenta) is shown (PDB 1QZ1). The sequence of plannexin is mapped in yellow. The figure was made using PyMOL Molecular Viewer (DeLano Scientific LLC. San Francisco, CA, USA).

We recently identified a peptide agonist of NCAM termed plannexin. Plannexin consists of a discontinuous sequence in the second NCAM Ig module that is predominantly located on one side of the C-terminal part of the module involved in homophilic *trans*-interactions [16] (Figure 1). Thus, plannexin is a synthetic compound that mimics a natural peptide sequence in NCAM.

**Figure 2:**
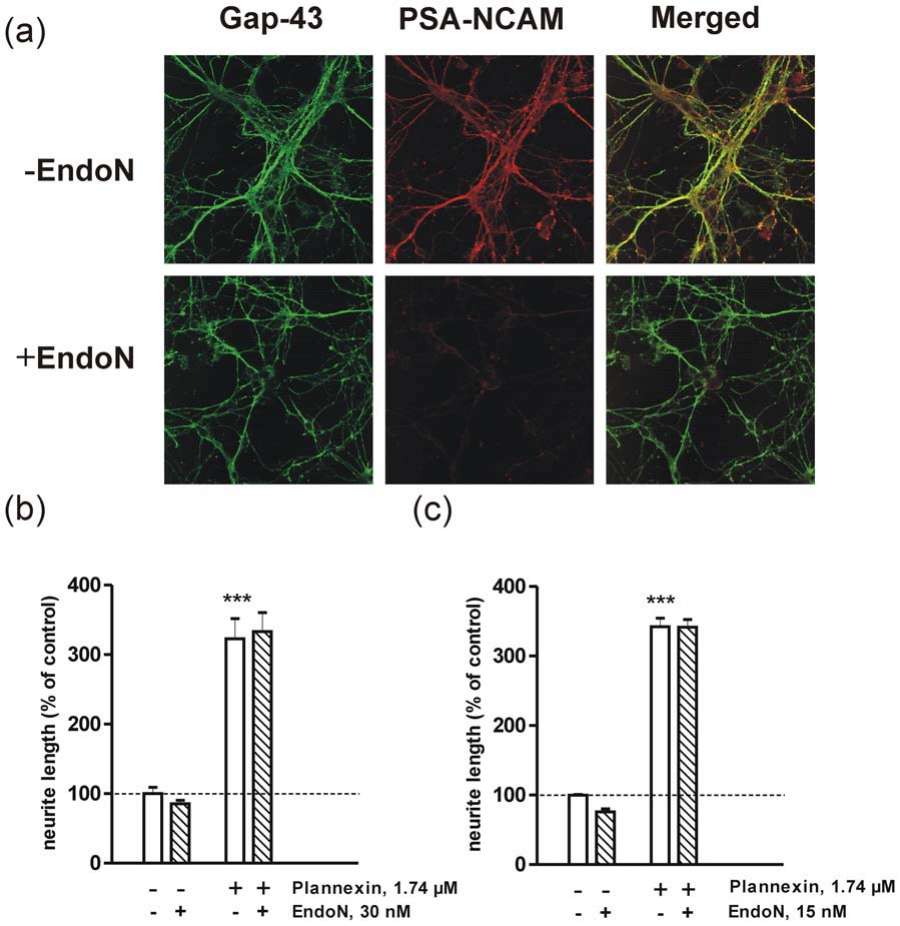
The effect of EndoN treatment on plannexin-induced neurite outgrowth and survival. (a) 125,000 cells/cm^2^ CGNs were left to differentiate for six days in the presence of high potassium (40 mM), followed by a 48 h incubation period with or without 15 nM EndoN. Cultures were double immunostained for GAP-43 (green) and PSA-NCAM (red). 12,500 cells/cm^2^ Hippocampal neurons (b) and CGNs (c) were grown for 24 h in the presence or absence of 1.74 µM plannexin and treated with 30 nM EndoN (**b**) or 15 nM EndoN (**c**). Results from 4–5 experiments are expressed as a percentage ± SEM, with unstimulated controls set at 100%. ***p<0.001 vs. untreated controls; ^+++^ *p*<0.001 vs. peptide-treated cultures without EndoN treatment.

Plannexin has been shown to be a potent inducer of neuritogenesis in primary CGNs [16]. Because primary neurons obtained from both CGNs and hippocampal neurons express highly polysialylated NCAM [28] (Fig. 2a], we investigated whether the neuritogenic effect of plannexin depends on NCAM polysialylation. PSA is a long, linear homopolymer of α-2,8-linked sialic acid. In vertebrates, PSA is added to the fifth Ig module of NCAM by two polysialyltransferases, ST8SiaII and ST8SiaIV. The PSA moiety is highly hydrated, and its enormous excluded volume is known to modulate NCAM-mediated adhesion and plasticity [29]. Hippocampal neurons (Fig. 2b) and CGNs (Fig. 2c) were treated for 24 h with plannexin. The cultures were fixed and immunostained for GAP-43, and neurite outgrowth was analyzed. Plannexin induced prominent neurite outgrowth in both types of primary neurons (hippocampal, Fig. 2b: F_3,16_ = 79.4; P<0.0001; CGNs, Fig. 2c: F_3,12_ = 290.7; P<0.0001). Next PSA was removed from NCAM by treating cultures with plannexin and EndoN in parallel. Plannexin's neuritogenic effects persisted in the presence of EndoN and thus do not depend on NCAM polysialylation.

### Plannexin enhances excitatory transmission in hippocampal area CA1

**Figure 3:**
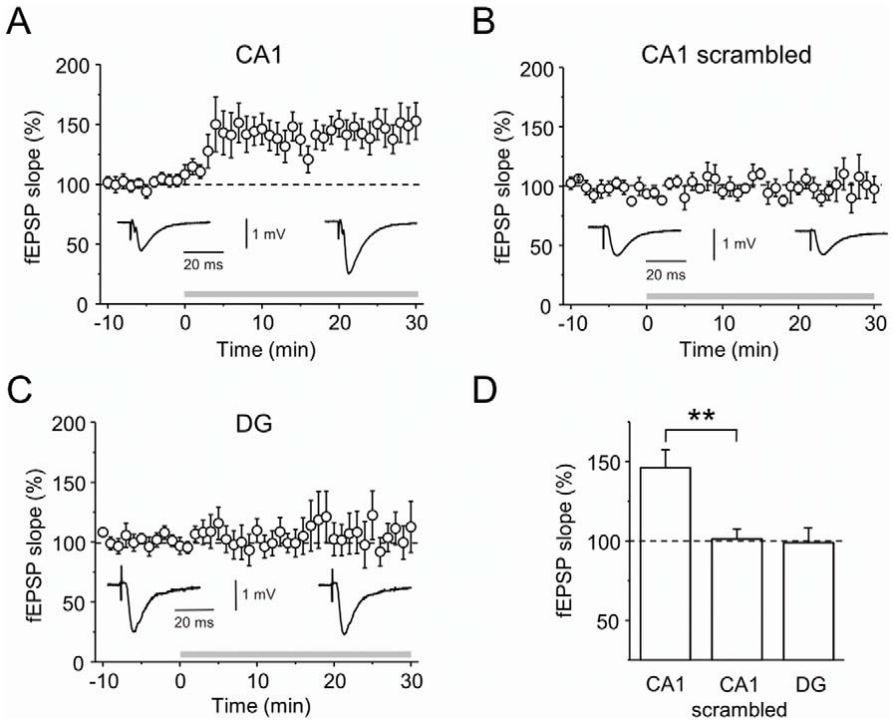
Plannexin enhances synaptic transmission of the Schaffer collateral pathway. A) Bath application of 10 µg/ml (i.e., 1.94 µM) plannexin increased the slope of the fEPSP evoked by Schaffer collateral stimulation (n = 17). B) A scrambled variant of plannexin did not affect the fEPSP (n = 5). C) Plannexin did not modulate the slope of the fEPSPs evoked by perforant pathway stimulation (n = 8). D) Summary of A–C, ** *p*<0.01.

Because plannexin affected neuronal connectivity in culture, we next asked if plannexin could modify synaptic transmission *in situ* in the hippocampus (Figure 3). Indeed, bath application of plannexin (10 µg/ml for 30 min; concentration: 1.94 µM) increased the slope of fEPSPs evoked by Schaffer collateral stimulation by 46.1±11.4% (p<0.001, one-population Student's t-test, n = 17, Figure 3A), and this effect was largely reversed within ∼30 min of washout. A scrambled version of plannexin did not alter the fEPSP slope using the same protocol (Figure 3B, +1.4±6.0%, n = 5, P = 0.83, one-population Student's t-test). However, plannexin had no significant effect on fEPSPs evoked in dentate gyrus by perforant pathway stimulation (Figure 3C, −1.0±9.0%, n = 8, P = 0.91). This disparity could be explained by a reportedly substantial difference in the polysialylation homeostasis between the two areas (see Discussion). Our results indicate therefore that plannexin couldacutely potentiate synaptic transmission in CA1 in a pathway-specific manner(whereas its scrambled variant is ineffective; Figure 3D, scrambled vs. plannexin, P<0.01, two-population unpaired Student's t-test).

### Plannexin improves spatial learning

**Figure 4:**
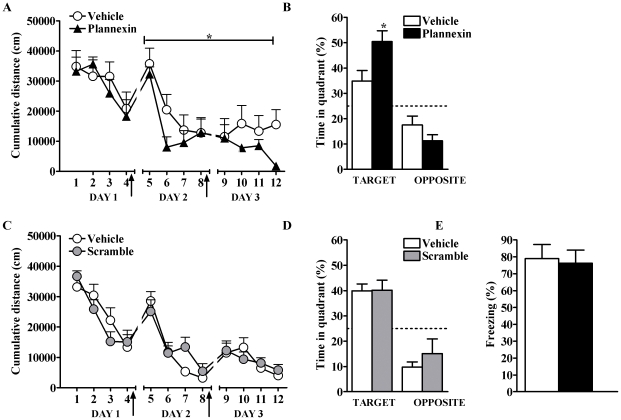
(A) Plannexin i.c.v. infusion immediately after the first and second training session (arrows) improves water maze learning (A, * *p*<0.05). (B) In the probe trial on day 3, rats showed better memory retention for the platform location compared to the control group (* *p*<0.05). (**C**) A scramble peptide infused i.c.v. immediately after the first and second training session (arrows) does not alter water maze learning as compared to vehicle-treated rats. (D) In the probe trial on day 3, rats infused i.c.v with a scramble peptide showed similar memory retention for the platform location compared to the vehicle group. N = 10/group. (**E**) Plannexin infusion did not affect a hippocampus-independent memory task, acoustic fear conditioning. No difference was detected in freezing levels between plannexin and control groups during the post-shock period at training and during tone presentation of the 24 h test. N = 8/group. Results are expressed as mean ± SEM.

In view of the facilitating effects of plannexin on synaptic transmission in the hippocampus, we next addressed its potential ability to influence spatial learning *in vivo* using the water maze [30]. As in a previous study in which the NCAM mimetic peptide FGL potentiated spatial memory [11], we evaluated the effect of post-training administration of the synthetic peptide on the learning and retrieval of this task. We trained rats for 3 consecutive days in daily sessions involving 4 trials each. Immediately after training on days 1 and 2, rats were injected i.c.v. with 2.5 µg of plannexin (n = 9) or control solution (either vehicle or scramble peptide, n = 8; as no differences were found between vehicle- and scramble peptide-injected rats, Figures 4C and 4D, their data were collapsed into one control group). All groups exhibited similar latencies to find the platform during the first training session (i.e., before any drug treatment was given; Figure 4A, day 1). Two-way ANOVA of the animals' cumulative distance from the platform on days 2 and 3 revealed a main effect of treatment (Figure 4A; F_1,105_ = 5.01, p<0.05) and of trial (F_7,150_ = 6.67, p<0.01), but not a significant trial×treatment interaction (F_7,105_ = 0.86, n.s.). As shown in Figure 4A, plannexin-treated rats showed shorter cumulative distances to the platform than vehicle-treated rats (F_1,105_ = 5.01, p<0.05).

On day 4, a probe trial was given in which the animals were exposed to the pool after the platform had been removed. The percentage of time that they spent in the target quadrant (vs. opposite quadrant) was measured as an index of memory retention. ANOVA indicated significant effects of quadrant (F_1,15_ = 33.78, p<0.01) and treatment (F_1,15_ = 6.507, p<0.05) as well as an interaction between them (F_1,15_ = 5.012, p<0.05). As shown in Figure 4B, plannexin-treated animals spent more time in the target quadrant than controls, indicating that the peptide improved memory. No differences in swimming speed were observed between the two groups (data not shown).

To assess the specificity of the observed effects on spatial learning and memory, we set up a series of experiments to evaluate whether the same treatment would influence (i) learning processes that do not depend on the hippocampus and (ii) general behaviors related to emotion and locomotion. Thus, a new set of animals was injected i.c.v. with either plannexin (n = 8) or vehicle (n = 8) immediately after training in the auditory fear-conditioning task, which is known to require the amygdala but not the hippocampus [31], [32]. As shown in Figure 4E, conditioned freezing to the tone was not affected by plannexin treatment (Mann Whitney, U_14_ = 31.0, n.s.). A second set of animals was tested in both the EPM and the open-field test after two plannexin vehicle infusions were administered on the two days preceding the tests. In the EPM, plannexin influenced neither the percent time spent in the open arms (vehicle: 6.05%±3.48; plannexin: 8.73±2.4%; n.s.) nor the total distance moved (vehicle: 38.50±4.6 m; plannexin: 40.71±1.8 m; n.s.). In the open field, plannexin affected neither the animals' latency to enter the center nor the percent time they spent in the center of the field (vehicle: 289.2±73.06 s and 2.16±0.53%; plannexin: 306.8±81.9 s and 1.78±0.2%, respectively; n.s.)

**Figure 5:**
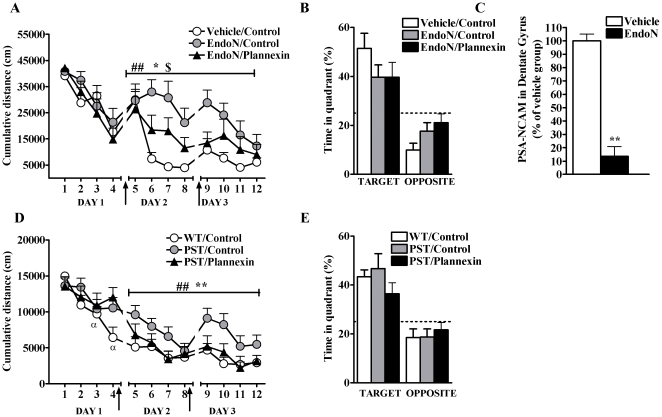
(A) Enzymatic removal of PSA-NCAM with EndoN i.c.v. infusion 24 h before training disrupts water maze acquisition on days 2 and 3 (p<0.05), and post-training (on day 1 and 2) plannexin injection restores the performance of EndoN pre-treated rats to control levels (##p<0.01, EndoN/Plannexin vs. EndoN/Control; *p<0.05 EndoN/plannexin vs. Vehicle control; $$p<0.01 EndoN/Control vs Vehicle Control, N = 9/group). (B) In the probe trial on day 3, no significant differences were detected between the three treatment groups. N = 9/group. (C)EndoN induces cleavage of PSA- NCAM in the dentate gyrus. Data are expressed as a percentage of the control group (vehicle-injected), **p<0.01 versus control group. N = 5–6/group. (D) PST mice, showing a genetic deficit in PSA-NCAM expression, are impaired in water maze learning (^α^p<0.05 vs trial 1). Plannexin treatment (arrows) restored water maze learning in PST mice (PST/Control vs. WT/Control ** *p*<0.01, PST/Plannexin vs PST/Control ## *p*<0.01 and PST/Plannexin vs. WT/control n.s.). N = 11–17/group. (E) In the probe trial on day 3, no significant differences were detected between PST and WT littermates or between PST control and PST plannexin-treated subjects. N = 10–11/group. Results are expressed as mean ± SEM.

Because our cell culture experiments showed that plannexin facilitates neurite outgrowth independently of the presence of PSA-NCAM, we set up a series of experiments to evaluate whether plannexin's facilitating effects on learning and memory could be observed under conditions of reduced PSA expression. As confirmed by repeated measures ANOVA, plannexin treatment of EndoN-infused rats following the same schedule as in former experiments (i.e., peptide administration immediately after training on days 1 and 2) partially restored spatial learning in Endo-N-treated animals to control levels (Figure 5A, main effect of treatment F_2,168_ = 6.65, p<0.01; Bonferroni post-hoc tests: EndoN vs. control p<0.01; EndoN/plannexin vs. EndoN/control, p<0.01; EndoN/plannexin vs. vehicle/control, p<0.05). Note that the lack of effect of Endo N in the first training day is in agreement with previous studies that showed that the same treatment affected memory consolidation and retrieval, but not initial acquisition, in the water maze (Venero *et al*., 2006). In the probe test on day 4, all groups swam significantly longer in the platform quadrant than in the opposite quadrant (main effect of quadrant F_1,24_ = 29.61, p<0.01), but no effect of treatment or quadrant×treatment interaction was found (n.s.: Figure 5B). Then, we verified that EndoN induces PSA cleavage under our experimental conditions. EndoN or vehicle was infused 4 days before animals were sacrificed. EndoN infusion reduced PSA-NCAM immunoreactivity in the hippocampus to 13.64±7.25% of control levels (Mann Whitney U_11_ = 6.0, p<0.01, Figure 5C).

To further confirm that plannexin can improve cognitive abilities under conditions of impaired PSA-NCAM expression, we evaluated whether it affected water maze learning in polysialyltransferase-1 (PST) KO mice. PST is the predominant enzyme that catalyzes the attachment of long sialic acid chains to NCAM in the postnatal brain [33]. As shown in Figure 5D, analysis of the performance on day 1, before vehicle or plannexin treatment, confirmed previous evidence of learning impairment in PST mice, although there was no significant genotype/treatment main effect, the genotype/treatment×trial interaction was significant (F_6,42_ = 2.24, p<0.05) and only WT group displayed efficient learning, swimming shorter cumulative distance to the platform on trial 3 and 4 compared to trial 1 (p<0.05), On day 2 and 3 repeated measures ANOVA confirmed a significant group effect on learning (F_2,42_ = 5.97, p<0.01), with plannexin infusion restoring PST KO mice performance to WT levels (PST/control vs. WT/control p<0.01, PST/plannexin vs. PST/control p<0.01 and PST/plannexin vs. WT/control n.s.). However, no differences among the groups were found for performance in the probe test (Figure 5E; n.s.), with all groups swimming in the target quadrant significantly longer than in the opposite quadrant, indicating that they remembered the location of the platform (p<0.01).

### Plannexin increases the percentage of mushroom spines in the CA1 hippocampal region

**Figure 6:**
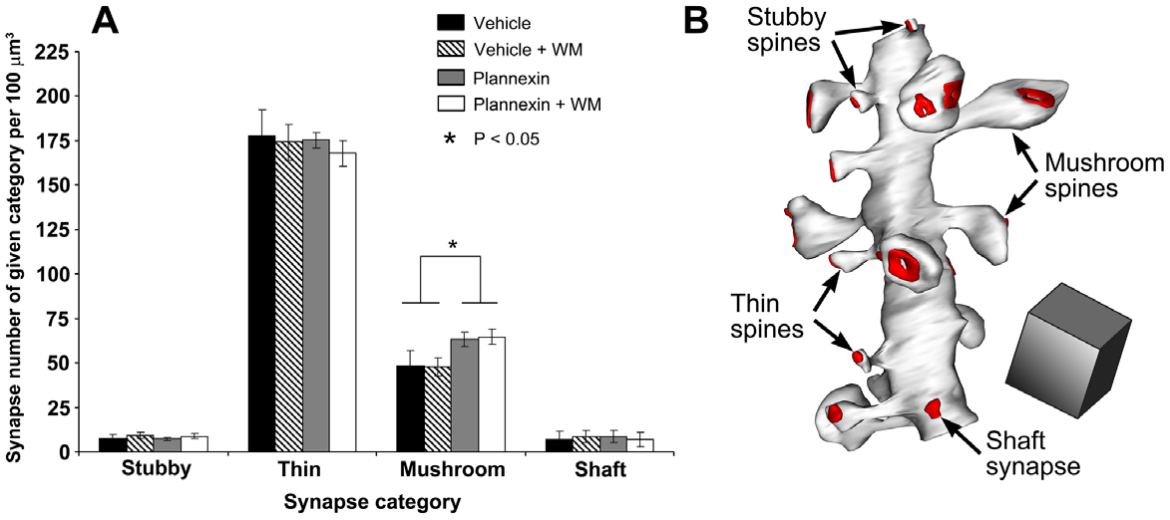
(A) Distribution of dendritic spine categories and distribution of synapses according to their spatial localization. The number of mushroom spines (expressed as synapse density per 100 µm^3^) increased significantly after plannexin treatment. (**B**) An example of a 3D reconstruction of a dendritic segment. Four categories of synapses are shown. Red color shows postsynaptic densities. Cube = 1 µm^3^. Results are expressed as mean ± SEM. * *p*<0.05.

Given that plannexin improved hippocampus-related learning and that electrophysiological experiments indicated that it potentiates synaptic transmission specifically in the hippocampal CA1 area, we investigated whether changes in synaptic structure could be observed in this region under conditions in which plannexin treatment was found to result in improved memory. Thus, a 2×2 experimental design was used to assess the effect of plannexin treatment on water maze training resulting in four experimental groups (n = 4/group): (i) vehicle: rats were i.c.v. infused with vehicle over 2 consecutive days and then left undisturbed until day 4, when animals were sacrificed; (ii) plannexin: same experimental design as in the previous condition, but the animals were infused with plannexin; (iii) vehicle+training: rats were trained for 3 days and injected with vehicle immediately after their training session on days 1 and 2; they were then sacrificed on day 4 after the probe test; (iv) plannexin+training: the same protocol as in the previous condition, except that the infusions included plannexin. The density of various categories of spines in these 4 groups are shown in Fig. 6A, together with a schematic description of the 3 spine types (mushroom, thin and stubby, plus shaft synapses). Figure 6B presents a 3-D reconstruction of a dendritic segment from over 100 serial ultrathin sections in CA1 *stratum radiatum*. Spines were categorized as thin, stubby and mushrooms spines (see Materials and Methods). A fourth category comprises synapses in which the presynaptic bouton contacts the dendrite directly and is termed a ‘shaft’ synapse. A 2×2 ANOVA indicated a significant effect of plannexin (F_1,12_ = 11.83, p<0.009) on the density of mushroom spines, but a lack of effect of water maze training (F_1,12_ = 0.07, p = 0.79) or for the interaction between the two factors (F_1,1_ = 0.25, p = 0.63). Thus, plannexin significantly increased the density of mushroom spines irrespective of water maze training. Further 2×2 ANOVAs performed on synapse density from the other spines categories failed to show an effect of plannexin or water maze training or their interaction [but note that a trend towards significance was found for plannexin treatment to reduce thin synapses (F_1,12_ = 4.57, p = 0.065)].

### Biochemical plasticity markers following plannexin treatment *in vivo*

As with NCAM, the neuritogenic effects of plannexin *in vitro* have been shown to be dependent on the activation of the FGFR [16]. Therefore, we evaluated whether plannexin treatment given *in vivo* was capable of activating FGFR1. Rats received an i.c.v. infusion of either plannexin or vehicle, and brains were dissected out 40 min after injection. Plannexin treatment was found to increase FGFR1 phosphorylation in CA1 homogenates compared to control-injected rats (0.94±0.04 and 0.80±0.03 arbitrary units, respectively; t = 1.996, df = 13, p = 0.036).

**Figure 7:**
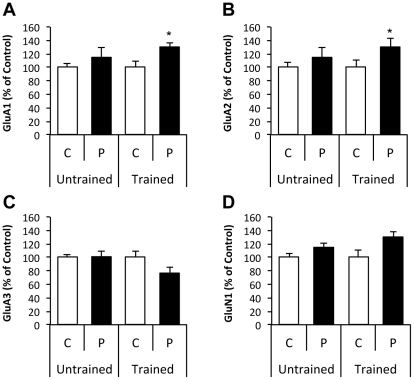
Synaptic expression of glutamate receptor subunits after plannexin treatment and water maze training: (A) GluA1 subunit; (B) GluA2 subunit; (C) GluA3 subunit; (D) GluN1B. Rats received 1 daily i.c.v. infusion of either vehicle or plannexin over 2 consecutive days either under basal conditions (untrained) or immediately after training in the water maze on days 1 and 2 (trained). Hippocampal samples were taken 2 days after the last drug infusion either under basal conditions (untrained) or following a 3^rd^ spatial training day and a probe trial just before sacrifice on day 4 (trained). Data are the mean ± SEM. *p<0.05 vs. controls. C = Control; P = Plannexin.

We also investigated whether plannexin and/or water maze training induced changes in glutamate receptor expression in the hippocampus at a time point when the behavioral effects of plannexin are particularly manifested (i.e., on experimental days 3–4 as shown in Figure 4), and the ultrastructural effects on different spine categories were also observed (i.e., on experimental day 4 as shown in Figure 6). Thus, the effect of plannexin treatment (2.5 µg i.c.v., administered on 2 consecutive days) on the expression of CA1 hippocampal synaptic AMPAR subunits (GluA1, GluA2 and GluA3; formerly GluR1, GluR2 and GluR3) and the GluN1 (formerly NR1) NMDA subunit was examined under 2 different experimental conditions (n = 9 per group): (i) Untrained: rats were sacrificed 2 days after the last plannexin injection under basal conditions; (ii) Trained: rats were trained for 3 days and injected immediately after training sessions on days 1 and 2; they were then sacrificed on day 4 immediately after the probe test. No difference between plannexin-treated and control animals was observed for any of the glutamate subunits assessed when they were examined under basal conditions (no training) (Figure 7A–D). In contrast, plannexin-treated rats submitted to a probe test on day 4 displayed enhanced synaptic expression of GluA1 (t = 2.77, df = 18, *p* = 0.01) and GluA2 (t = 2.82, df = 16, *p* = 0.01) in CA1 compared to controls (Figure 7A, B). There was a trend toward significance for GluA3 (t = 2.00, df = 16, *p* = 0.06), and levels from plannexin-treated rats were lower than controls following the probe test (Figure 7C). No differences in GluN1 expression between plannexin-treated and control animals were observed under either of the experimental conditions [untrained (t = 0.30, df = 16, *p* = 0.76); trained (t = 1.87, df = 16, *p* = 0.09), Figure 7D]. When the total expression of the different receptor subunits was analyzed in hippocampal homogenates, no differences were found between plannexin-treated and control groups (all n.s.; data not shown).

## Discussion

NCAM plays a key role in synaptic plasticity [2], [34], [35] and cognitive function [9], [36], [37], suggesting that it may be a particularly relevant target for which to develop cognition-enhancing drugs. However, NCAM is a structurally and functionally complex molecule with multiple domains engaged in a variety of actions ranging from cell–cell adhesion to cytoplasmic signaling [14]. These features raise the question as to which NCAM fragment should be targeted to improve cognition. In this study, we tested the recently newly synthesized peptide plannexin that mimics a homophilic trans-binding site in the NCAM Ig2 module (which binds to the NCAM Ig3 module [16]) for its ability to affect synaptic plasticity and memory formation. We found that plannexin facilitates neurite outgrowth in primary hippocampal neuronal cultures and improves spatial learning in rats, both under basal conditions and under reduced PSA-NCAM. The effect on spatial learning seems to be quite specific because plannexin affects neither hippocampus-independent learning, auditory fear conditioning (however, a note of caution should be added since recent work indicates that the amygdala's involvement in auditory fear conditioning takes place during acquisition, not consolidation, of information [38], and the present study only involved post-training peptide injections), nor emotional or locomotor responses to novelty. We also found that plannexin enhances hippocampal FGFR1 phosphorylation and synaptic transmission in CA1 (but not in the dentate gyrus), where it also increases the number of mushroom spines and the synaptic expression of the AMPAR subunits GluA1 and GluA2. Altogether, these results highlight plannexin as an important modulator of hippocampus-dependent cognition and synaptic plasticity.

Plannexin was previously shown by surface plasmon resonance analysis to bind the NCAM Ig3 module and to induce neurite outgrowth in primary cultures of CGNs and dopaminergic neurons in an NCAM-dependent manner [16]. Here, we extend these findings to primary hippocampal cultures. We show that the magnitude of the effect is comparable to that in CGN cultures, and in both cases, the effect is independent of PSA-NCAM. Importantly, we show that plannexin improves long-term spatial memory when injected icv in rats using a regimen of infusions (ie, treatment was given immediately after water maze training on days 1 and 2) previously proven to be effective for another NCAM-related peptide, FGL, which binds and activates FGFR [11]. The critical role of PSA-NCAM in synaptic plasticity and memory is well established in the literature [34], [35], [39]–[42]. Plannexin also improves learning and memory in PSA-NCAM-deficient animals: both in rats infused with the enzyme EndoN and in PST KO mice, which show reduced PSA-NCAM expression in the adult forebrain [40], [43] and deficiencies in learning during training days 2 and 3. This indicates again that plannexin's effect is independent of PSA-NCAM expression, and it underscores plannexin as an effective treatment for cognitive deficits associated with PSA-NCAM deficiency, a condition reported to accompany, for example, aging [44], [45].

When hippocampal slices were examined, synaptic transmission from Schaffer collaterals to CA1 pyramidal cells, but not at the perforant path synapses in the dentate gyrus, was potentiated following acute plannexin treatment. This rapid effect was paralleled by our *in vivo* data showing increased FGFR phosphorylation in the hippocampus 40 min after icv plannexin infusion. This activation of FGFR supports the view that plannexin effects include a previously well characterized pathway for NCAM actions in neural plasticity [46], [47]. These data agree well with former work in cell cultures showing that plannexin-induced neurite outgrowth is dependent on FGFR activation [16]. NCAM-mediated signaling includes direct binding to and activation of FGFR [48]. Importantly, plannexin was found to activate FGFR via its binding to NCAM [16], which differs from the mechanisms of action of the FGL peptide, whose plasticity-inducing and cognitive-enhancing effects are not dependent on the presence of NCAM [10], [48]. The absence of an effect of plannexin at the perforant pathway suggests that plannexin could affect NCAM/FGFR signaling differentially across synaptic populations. Indeed, different expression of the FGFR receptor subtypes across hippocampal subfields, specifically the absence of FGFR-2 and 3 mRNA in the dentate gyrus, has been documented [49]. Furthermore, that polysialylation of hippocampal NCAM persists into the adulthood particularly in the dentate gyrus [50]. This could suggest a dependence of acute plannexin effects to polysialylation although its long-term effects on neurite outgrowth observed here were insensitive to polysialylation.

In the CA1 *stratum radiatum*, plannexin-treated animals showed an increase in the percentage of mushroom spines when analyzed 2 days after the last peptide treatment. This time point was selected to coincide with the period at which facilitating effects of the peptide are evident (Figure 4). Interestingly, the effect of plannexin was detected in both water maze-trained and untrained animals, indicating that the observed structural changes do not require synergy between the peptide's actions and training-triggered activity. This is at odds with the enhanced synaptic expression of GluA1 and GluA2 subunits observed in the CA1 region in water maze-trained plannexin-treated animals but not in untrained ones. There are different interpretations of these data. The group simultaneously subjected to drug and training regimens has the ability to selectively drive these AMPAR subunits to the synaptic compartment (note that there were no changes in their total expression levels in homogenates); or this putative effect of plannexin does not require a history of prior training, but is triggered by the probe test given to trained animals in this experimental group just before samples are collected. The latter possibility seems the most plausible according to our unpublished experiments, in which we observed no changes in the levels of the different glutamate receptor subunits in a group of animals that followed the same treatment as in the present study except that samples were collected on day 4 under basal conditions following a probe test.

Plannexin treatment might, therefore, affect mechanisms involved in the synaptic trafficking of AMPAR subunits. Furthermore, it may be capable of driving GluA1 and GluA2 to the synaptic compartment under conditions of circuit stimulation, such as those induced by spatial testing in the water maze. The link of such a mechanism with the improvement in learning and memory found in plannexin-treated animals is strongly supported by a large body of data linking synaptic expression of these AMPAR subunits with facilitated synaptic plasticity [51]–[55] and memory [27], [53], [56], [57]. An important prediction derived from these data is that, given the appropriate behavioral stimulation 2 days after the last peptide infusion, animals in the plannexin-treated untrained group should show enhanced synaptic expression of GluA1 and GluA2 subunits as well as facilitated learning capabilities. This will be evaluated in future studies aimed at understanding the specific temporal and experimental dynamics required for plannexin's cognitive-enhancing properties. Although links between NCAM, AMPAR [58], [59] and NMDAR [60] function have only recently begun to be uncovered, early indirect observations also support this connection. NCAM-induced neurite outgrowth involves activation of both FGFR and Fyn, a member of the Src-family, as shown for plannexin in vitro [16]. Both signaling cascades have been shown, independently, to affect the expression of AMPARs [61], [62].

As to the structural changes observed, plannexin induced an increase in the number of mushroom spines, which have been called ‘memory’ spines [63], [64]. They are more stable over time than spines with small heads, and they normally show enhanced PSD complexity and/or dimensions after physiological stimulation [63], [64], as observed in our plannexin-treated animals. The PSDs of these spines normally contain a higher density of glutamate receptors, which also fits with the enhanced AMPAR expression induced by plannexin treatment in trained animals. In fact, the GluA2 subunit was shown to play a crucial role in the formation and growth of dendritic spines in cultured hippocampal neurons and to interact with the cell adhesion molecule N-cadherin to produce this effect [65]. Importantly, evidence was also presented that GluA2-containing receptors contribute to features of mushroom spines, with GluR2 KO mice showing lower numbers of synapses on mushroom spines and significant decreases in the volume and surface area of mushroom spines and their PSDs [66]. In addition, our ultrastructural observations also confirmed typical features of these mushroom spines, such as their higher endosome content [64]. The morphological changes induced by plannexin largely resemble those previously found after LTP induction both in vitro [67], [68] and in vivo [69], [70]. Interestingly, chronic treatment with the NCAM mimetic peptide FGL for 2.5 weeks in aged rats also leads to an increased proportion of mushroom spines [71]. Altogether, the reported morphological and biochemical findings are important because they show that plannexin treatment provides structural and molecular changes correlated with enhanced synaptic strength [72]. A strong memory is believed to require lasting morphological changes that increase the strength of the synapses encoding information related to memory and that increase the size of spine heads to accommodate more glutamate receptors [72], [73].

Altogether, this study presents compelling evidence that plannexin is an important facilitator of synaptic functional, structural and molecular plasticity in the hippocampal CA1 region, highlighting the fragment in NCAM's Ig3 module where plannexin binds as a novel target for the development of cognition-enhancing drugs.
